# Supplementary material for: Comparison of Single Cell Transcriptome Sequencing Methods: Of Mice and Men
Source: Genes (Basel). 2023 Dec 16;14(12):2226. doi: 10.3390/genes14122226 (PMC10743076; doi:10.3390/genes14122226)
Supplement: Supplementary file 1 [file genes-14-02226-s001.zip › Supplementary methods and results.pdf]

Appendix:

Comparison of Single Cell Transcriptome Sequencing Methods: Of Mice and Men

Bastian V. H. Hornung [1,2], Zakia Azmani [1,2], Alexander T. den Dekker [1,2], Edwin Oole [1,2], Zeliha Ozgur [1,2],
Rutger W. W. Brouwer [1,2], Mirjam C. G. N. van den Hout [1,2] and Wilfred F. J. van IJcken [1,2,*]

1 Department of Cell Biology, Erasmus University Medical Center Rotterdam, Wytemaweg 80, 3015CN Rotterdam, The Netherlands
2 Genomics Core Facility, Erasmus University Medical Center Rotterdam, Wytemaweg 80, 3015CN Rotterdam, The Netherlands
* Correspondence: w.vanijcken@erasmusmc.nl

Supplementary methods

Culturing of K562 cells

K562 cells (ATCC CCL-243) were maintained at 37°C under 5% CO_2 in RPMI medium supplemented with 10% FBS and penicillin–streptomycin.

Culturing of mouse embryonic stem cells

Mouse embryonic stem cells (mESC) were cultured as previously described [1]. Mouse ESCs were cultured on 0.1% gelatin and FCS-coated plates in N2B27 medium supplemented with 1000 U/ml LIF, 3 µM CHIR99021 (Axon Medchem) and 2 µM PD0325901 (Merck). Plates were coated by brief incubation of the wells with 0.1% gelatin in PBS, followed by 1h incubation with FCS at 37 °C. Before culture, plates were rinsed with PBS. N2B27 medium consisted of DMEM/F12 combined with Neurobasal medium (1:1 ratio) supplemented with 1% B27 supplement, 0.5% N2 supplement, 50 µM β-mercaptoethanol, 2mM Glutamax, 0.033% BSA 7.5% solution, 100 U/ml penicillin and 100ug/ml streptomycin (all from Invitrogen). Cells were cultured at 5% CO2 and 37 °C, passaged 1:5 every 2-3 days by trituration of colonies to a single cell suspension using 0.05% trypsin-EDTA. Trypsin was quenched using soybean trypsion inhibitior (Sigma). Cells were routinely tested for mycoplasma contamination every 2-3 months. R1 cells, obtained from Stanford Transgenic Facility, were used. Cells were harvested at different passaging stages.

Plexwell

The RNA-Seq library was prepared by using the manufacturer's prototcol: plexWell™ Rapid Single Cell protocol (seqWell Inc.) v20210205 at half the indicated volumes.

Briefly, cells were harvested and put into PBS (degassed) as single cell suspension. Single cells were isolated using the CellenOne instrument (SCIENION GmbH), dispensing 1 single cell into each well of the plexWell™ lysisplate. In one case the dispense was performed in a checkerboard pattern (alternating K562 and mESC), in one case the cells were dispensed in a row-based pattern. After dispense the lysis plate was stored at -80C.

Lysis was done at 10 min at 72°C, hold 4°C, followed by cDNA generation and amplification using PCR: 30 min at 50°C, 3 min at 98°C, 19-21 cycles of (20 s at 98°C, 20 s at 67°C, 3 min at 72°C), hold 4°C.

cDNA quantification was done using the Quant-iT™ dsDNA Assay Kit, high sensitivity (Thermo Fisher Scientific Inc.) and cDNA quality was checked with the 2100 Bioanalyzer (Agilent Technologies Inc.) using a DNA1000 High Sense assay.

cDNA was normalized to 250 pg/uL. Because of the half reaction volumes, only 500 pg was used as input into the next step. Sample barcoding was done by tagmentation, followed by pooling of the individual wells. This pool was purified and tagmented with the Pool Barcode. The Pool-barcoded sample was purified and amplified by PCR: 10 min at 72°C, 3 min at 95°C, 12 cycles of (30 s at 98°C, 15 s at 68°C, 30 sec at 72°C), 3 min at 72°C, hold 4°C. When the PCR is finished a final purification was performed.

Library quantification and quality control was done with the 2100 Bioanalyzer (Agilent Technologies Inc.) using a DNA1000 High Sense assay.

For the paired-end dataset, the wells were divided into four sets, of which one was processed with the full amount of reagents, one with 50% of the reagents, one with 20%, and one with 10%.

Smart-seq3

This workflow, derived from [2], follows the obligatory steps in the protocol under [3], although with partially adjusted concentrations.

Lysis buffer was dispensed in single wells of a 384-well plate (Eppendorf) with the Mantis (Formulatrix), and the plate was sealed and stored at −20°C until needed.

The Smart-seq3 lysis buffer was composed of 0.4 µl Poly-ethylene Glycol 8000 (50% solution w/v, Sigma-Aldrich), 0.06 µl Triton-X100 (10% v/v, Sigma-Aldrich), 0.04 µl RNAse inhibitor (40 U/µl (RRI), Takara), 0.02 µl Smart-seq3 oligo-dT primer 100 µM (5′-biotin-ACGAGCATCAGCAGCATACGA T30VN-3′; IDT), 0.08 µl dNTP mix (25 mM each, Invitrogen), and water to 3 µl final volume dispensed with the Mantis (Formulatrix).

K562 and mESC cells were dispensed with the CellenOne instrument (SCIENION GmbH) into 384-well plates (Eppendorf) containing 3 µl of lysis buffer per well.
The plates were sealed, spin down immediately after dispense and stored at −80 °C.

Plates were removed from the −80 °C storage and centrifuge at 1500rpm (400g) for 1 min, transferred to a preheated Thermocycler (Westburg), incubated for 10 min at 72 °C and immediately placed on ice.
Next, 1 µl of reverse transcription mix, containing 0.10 µl Tris-HCL, pH 8.3 1 M (Sigma-Aldrich), 0.12 µl NaCl 1 M (Ambion), 0.04 µl GTP 100 mM (Thermo Fisher Scientific Inc.), 0.10 µl MgCl2 100 mM (Ambion), 0.32 µl DTT 100 mM (Thermo Fisher Scientific Inc.), 0.05 µl RRI 40 U/µl (Takara), 0.08 µl of Smart-seq3 TSO 100 µM; 5′-biotin-AGAGACAGATTGCGCAATGNNNNNNNNrGrGrG-3′; Biolegio) and 0.04 µl of Maxima H-minus reverse transcriptase enzyme 200 U/µl (Thermo Fisher Scientific Inc.), and water to 1 µl final volume was added to each sample with the Mantis (Formulatrix) and transferred to a preheated Thermocycler (Westburg). Reverse transcription and template switching were carried out at 42 °C for 90 min followed by 10 cycles of 50 °C for 2 min and 42 °C for 2 min. The reaction was terminated by incubating at 85 °C for 5 min.

PCR pre-amplification was performed directly after reverse transcription by adding 6 µl per well of PCR mix, bringing reaction concentrations to 5 µl KAPA HiFi PCR buffer (2x) (Roche), 0.05 µl Smart-seq3 forward PCR primer 100 µM (5′-TCGTCGGCAGCGTCAGATGTGTATAAGAGACAGATTGCGCAATG-3′; Biolegio) and 0.01 µl Smart-seq3 reverse PCR primer 100 µM (5′-ACGAGCATCAGCAGCATACGA-3′; Biolegio) and water to 6 µl final volume. PCR was cycled as follows: 3 min at 98 °C for initial denaturation, then *N* cycles of (20 s at 98 °C, 30 s at 65 °C and 4 min at 72 °C. Final elongation was performed for 5 min at 72 °C), 15 °C on hold. The number of cycles depended on the cell type.

Samples were cleaned up using 6 µl per well AMpure XP beads (Beckman Coulter) and eluted in 12 µl of nuclease free water by using the Mantis (Formulatrix).

In order to assess cDNA size distribution, 11 samples from each plate were randomly chosen, loaded on a High Sensitivity DNA chip and run on a 2100 Bioanalyzer System (Agilent Technologies Inc.) and all cDNA concentrations were quantified using the Quant-iT™ dsDNA Assay Kit, high sensitivity (Thermo Fisher Scientific Inc.).

Samples were diluted to a final cDNA concentration of 200 pg/µl based on the PicoGreen measurements.

The cDNA libraries were normalized to 200 pg/µl for the tagmentation. Transfer 2 µl of cDNA to a new 384 well plate with the Mosquito (Sptlabtech). Two microliters Tagmentation was carried out with the I.DOT (Dispendix), consisting of 4x Tris tagmentation buffer (20 µl Tris-HCl 1M, pH 7.5, 100µl MgCl2 100 mM, 100 µl DMF), 16.9 µl TDE1 (Illumina Tagment DNA Enzyme and Buffer).
Plate was transferred to a preheated Thermocycler (Westburg) incubated at 55 °C for 10 min, the Tn5 was inactivated with 1 µl 0.2% Sodium Dodecyl Sulfate (SDS) buffer with the I.DOT (Dispendix), followed by 5 min incubation at room temperature.

PCR mix 7.5 µl per well, consisting (2.80 µl Phusion HF Buffer 5x (Thermo Fisher Scientific Inc.), 0.060 µl Phusion DNA polymerase 2 U/µl (Thermo Fisher Scientific Inc.), 0.120 µl dNTP 25 mM (Invitrogen) and 4.520 µl nuclease-free water to 4 µl final volume was added with the I.DOT (Dispendix) to each well and using the Mosquito (Sptlabtech) 1.5 µL 1µM I5/I7 index mix was transferred to the sample wells.
The enrichment PCR reaction was done at 72 °C for 3 min, 98 °C for 3 min and then 12 cycles of (98 °C for 10 s, 55 °C for 30 s, 72 °C for 30 s), 72 °C for 5 min, 4 °C hold in a preheated Thermocycler (Westburg).

After PCR, samples were pooled, 1 µl per well using the Mosquito (Sptlabtech) and cleaned up with a ratio of 0.6:1 Ampure XP beads (Beckman Coulter) and eluted in 15 µl of Resuspension Buffer.
Library size distributions were checked on a high-sensitivity DNA chip with the 2100 Bioanalyzer (Agilent Technologies Inc.).

Smart-seq3 Libraries were sequenced on the HiSeq2500 or Nextseq2000 (Illumina) platform.

FLASH-seq

The FLASH-seq workflow[4] is derived from the Smart-seq2 workflow and in most parts either identical or similar, and furthermore mostly identical to the published protocol under [5].
Lysis buffer was dispensed in single wells of a 384-well plate (Eppendorf) with the Mantis (Formulatrix), and the plate was sealed and stored at −20°C until needed.

The FLASH-seq lysis buffer was composed of 0.02 µl Triton-X100 (10% v/v, Sigma-Aldrich), 0.24 µl dNTP mix (25 mM each, Invitrogen), 0.018 µl FS-dT30VN (5'Bio-AAGCAGTGGTATCAACGCAGAGTACT30VN-3' (Bio, biotin); 100 µM, Biolegio), 0.03 µl RNAse inhibitor (40 U/µl, Takara), 0.012 µl dithiothreitol (100 mM, Invitrogen), 0.2 µl betaine (5 M, Sigma-Aldrich), 0.09 µl dCTP (100 mM, Invitrogen), 0.092 µl FS-TSO (5'Bio-AAGCAGTGGTATCAACGCAGAGTACrGrGrG-3' (Biolegio); 100 µM, IDT), and water to 1 µl final volume dispensed with the Mantis (Formulatrix).

For 3 datasets the K562 and mESC cells were dispensed with the CellenOne instrument (SCIENION GmbH) into 384-well plates (Eppendorf) containing 1 µl of lysis buffer. For one dataset the dispense was performed with an F.SIGHT single cell sorter (Cytena). In two cases the cells were dispensed in a checkerboard pattern (alternating K562 and mESC), in one case only K562 were dispensed, and in one case the cells were dispensed row-wise.
The plates were sealed, spin down immediately after dispense and stored at −80 °C.

Plates were removed from the −80 °C storage and centrifuge at 1500rpm (400g) for 1 min, transferred to a preheated Thermocycler (Westburg), incubated for 3 min at 72 °C and immediately placed on ice.
Next, 4 µl of reverse transcription-PCR mix was added using the I.DOT (Dispendix). Reverse transcription-PCR mix composition: 0.238 µl dithiothreitol (100 mM, Invitrogen), 0.8 µl betaine (5 M, Invitrogen), 0.046 µl magnesium chloride (1 M, Ambion), 0.096 µl RNAse inhibitor (40 U/µl, Takara), 0.05 µl Superscript IV (200 U/µl, Invitrogen), 2.5 µl KAPA HiFi Hot-Start ReadyMix (2x, Roche) and nuclease-free water to 4 µl final volume.
The following RT-PCR program was used: 60 min at 50 °C, 98 °C for 3 min, then *N* cycles of (98 °C for 20 s, 67 °C for 20 s, 72 °C for 6 min) 15 °C on hold. The number of cycles depended on the cell type.

Samples were cleaned up using 8 µL/well AMPure XP beads (Beckman Coulter) and eluted in 15 µl of nuclease free water by using the Mantis (Formulatrix).

In order to assess cDNA size distribution, 11 samples from each plate were randomly chosen, loaded on a 2100 Bioanalyzer (Agilent Technologies Inc.) using a DNA1000 High Sense assay and all cDNA concentrations were quantified using the Quant-iT™ dsDNA Assay Kit, high sensitivity (Thermo Fisher Scientific Inc.).

Samples were diluted to a final cDNA concentration of 200 pg/µl based on the Quant-iT measurements.

The cDNA libraries were normalized to 200 pg/µl for the tagmentation. Transfer 2 µl of cDNA to a new 384 well plate with the Mosquito (Sptlabtech). Two microliters Tagmentation was carried out with the I.DOT (Dispendix), consisting of 4x Tris tagmentation buffer (20 µl Tris-HCl 1M, pH 7.5, 100µl MgCl2 100 mM, 100 µl DMF), 16.9 µl TDE1 (Illumina Tagment DNA Enzyme and Buffer).

Plate was transferred to a preheated Thermocycler (Westburg) incubated at 55 °C for 10 min, the Tn5 was inactivated with 1 µl 0.2% Sodium Dodecyl Sulfate (SDS) buffer dispensed with the I.DOT (Dispendix), followed by 5 min incubation at room temperature.
The tagmentation steps and thermocycler programme have been adjusted and are not identical to the ones in the published workflow, deviating in time, exact enzyme and reagent composition.

PCR mix 7.5 µl per well, consisting (2.80 µl Phusion HF Buffer 5x (Thermo Fisher Scientific Inc.), 0.060 µl Phusion DNA polymerase 2 U/µl (Thermo Fisher Scientific Inc.), 0.120 µl dNTP 25 mM (Invitrogen) and 4.520 µl nuclease-free water to 4 µl final volume was added with the I.DOT (Dispendix) to each well and using the Mosquito (Sptlabtech) 1.5 µL 1µM I5/I7 index mix was transferred to the sample wells.

The enrichment PCR reaction was done at 72 °C for 3 min, 98 °C for 3 min and then 12 cycles of (98 °C for 10 s, 55 °C for 30 s, 72 °C for 30 s), 72 °C for 5 min, 4 °C hold in a preheated Thermocycler (Westburg).

After PCR, samples were pooled, 1 µL per well using the Mosquito (Sptlabtech) and cleaned up with a ratio of 0.8:1 Ampure Beads (Beckman Coulter) and eluted in 15 µl of Resuspension Buffer.

The concentration and quality of the cDNA and sequencing library were determined with the 2100 Bioanalyzer (Agilent Technologies Inc.) using a DNA1000 High Sense assay.

FLASH-seq Libraries were sequenced on the NextSeq 2000 (Illumina).

For one of the FLASH-seq datasets half of the wells contained the Protector RNAse inhibitor from Sigma Aldrich, the other half contained the RNAse inhibitor RRI 2313A from Takara.

10X

ScRNA-Seq library was prepared by using the Chromium Next GEM Single Cell 3’ protocol v 3.1 (CG000315 Rev C) (10x Genomics).

Briefly, targeted cell recovery was aimed at 5000 single cells. Gel Bed-in-Emulsions (GEMs) were generated with the Chromium Controller (10x Genomics).

The concentration and quality of the cDNA and sequencing library were determined with the 2100 Bioanalyzer (Agilent Technologies Inc.) using a DNA1000 High Sense assay.

The library was sequenced on an NextSeq2000 sequencer (Illumina). Paired-end clusters were generated with 28 bases in read 1 (containing cell barcode and UMI), 90 bases in read 2 (containing the transcriptome read) and with dual 10bp indices.

SORT-seq

K562 and mESC cells were dispensed with the CellenOne instrument (SCIENION GmbH) into 384-well plates called cell capture plates, that were ordered from Single Cell Discoveries, a single-cell sequencing service provider based in the Netherlands. Each well of a cell-capture plate contains a small 50 nl droplet of barcoded primers and 10 µl of mineral oil (Sigma-Aldrich M8410). After sorting, plates were immediately spun and placed on dry ice. Plates were stored at -80°C. Plates were shipped on dry ice to Single Cell Discoveries, where single-cell RNA sequencing was performed according to an adapted version of the SORT-seq protocol [6] with primers described in [7]. Cells were heat-lysed at 65°C followed by cDNA synthesis. After second-strand cDNA synthesis, all the barcoded material from one plate was pooled into one library and amplified using in vitro transcription (IVT). Following amplification, library preparation was done following the CEL-Seq2 protocol [8] to prepare a cDNA library for sequencing using TruSeq small RNA primers (Illumina). The DNA library was paired-end sequenced on an Illumina Nextseq™ 500, high output, with a 1×75 bp Illumina kit (read 1: 26 cycles, index read: 6 cycles, read 2: 60 cycles).

VASA-seq

K562 and mESC cells were dispensed with the CellenOne instrument (SCIENION GmbH) into 384-well plates called cell capture plates, that were ordered from Single Cell Discoveries, a single-cell sequencing service provider based in the Netherlands. Each well of a cell-capture plate contained a small 25 nL droplet of barcoded primers and 5 µL of mineral oil (Sigma-Aldrich M8410). After sorting, plates were immediately spun and placed on dry ice. Plates were stored at -80°C.

Plates were shipped on dry ice to Single Cell Discoveries, where single-cell RNA sequencing was performed according to an adapted version of the VASA-seq protocol [9]. Cells were enzymatically lysed, followed by fragmentation, end repair, poly(A)-tailing, reverse transcription, and second-strand synthesis. After second-strand synthesis, all the barcoded material from one plate was pooled into one library and amplified using in vitro transcription. Further, ribosomal aRNA was depleted. After depletion, adapter ligation, reverse transcription, and indexing PCR were performed to prepare a cDNA library. The cDNA library was paired-end sequenced on an Illumina Nextseq™ 500, high output, with a 1×75 bp Illumina kit (read 1: 26 cycles, index read: 6 cycles, read 2: 60 cycles).

HIVE

Cells were measured on a countess2, and were afterwards diluted to acquire 15,000 cells for the human and mouse sample. The two samples containing both cells was mixed based on the measurements of both separate samples.
All HIVEs were processed according to the manufacturer's instructions with the HIVE scRNAseq v1 Processing Kit User Protocol - Revision A, v22.09 from September 2022. During the processing the HIVE with the mixed cells it was noted that the frozen liquid did not completely

cover the HIVE array, but processing was still continued. One of the four HIVEs, containing both human and mouse cells, was stored at -20°C, according to the manufacturer's instructions, for approximately six months.
For the HIVE CLX the user protocol v 1.0 from May 2023 was followed, and 30,000 cells were loaded. Cells were measured with the Nexcelom Cellometer. Three samples were directly processed, and one sample was stored. During the preparation of the long-term sample an issue appeared, during the incubation with the filter plate, the well was nearly empty, although it cannot be said if this leakage affected the sample for a long time. The long-term HIVE CLX sample was stored at -80°C, according to the manufacturer's instructions, for approximately 6 weeks. Both the frozen HIVE and HIVE CLX were processed at the same time.

Evercode WT Mini

The instructions of the Evercode WT Mini manual V2.1.2 from August 2023 were followed, with the alternative version of the Evercode WT Mini kit, including SX200 instead of SX100. Less mESC cells were used than intended, because not enough could be obtained due to laboratory variation. During the cell fixation step, the centrifuge was spun at 300 x g instead of the recommended 200 for the lower amount of mESC cells, and only 80 µl were used instead of 150. The higher rotation speed was retained in later steps. No straining was performed for these samples. During the barcoding, the mixture of the lysis mix was adjusted to 50% more, to 82.5 µl lysis buffer instead of 55 µl, 16.5 µl lysis enzyme instead of 11, and a 3 ml tube was used instead of a 1.5 ml tube. At the end three sublibraries were created, of which two were sequenced.

Bulk

RNA was isolated with the RNEasy plus Micro kit by Quiagen, with 500.000 cells per sample. Total RNA for K562 and mESC triplicates were checked for quality on a Agilent Technologies 2100 Bioanalyzer using a RNA nano assay. Triplicate RNA-Seq libraries were prepared according to the Illumina TruSeq stranded mRNA protocol (www.illumina.com). Briefly, 200 ng of total RNA was purified using poly-T oligo-attached magnetic beads to end up with poly-A containing mRNA. The poly-A tailed mRNA was fragmented and cDNA was synthesized using SuperScript II and random primers in the presence of Actinomycin D. cDNA fragments were end repaired, purified with AMPure XP beads, A-tailed using Klenow exo-enzyme in the presence of dATP. Paired end adapters with dual index (Illumina) were ligated to the A-tailed cDNA fragments and purified using AMPure XP beads. The resulting adapter-modified cDNA fragments were enriched by PCR using Phusion polymerase as followed: 30 s at 98°C, 15 cycles of (10 s at 98°C, 30 s at 60°C, 30 s at 72°C), 5 min at 72°C. PCR products were purified using AMPure XP beads and eluted in 30 µl of resuspension buffer. One microliter was loaded on an Agilent Technologies 2100 Bioanalyzer using a DNA 1000 assay to determine the library concentration and for quality check. A part of the workflow was automated with the Bravo automated liquid handling platform (Agilent Technologies Inc.).

The sequencing libraries were pooled together to get a stock of 2 nM. Loading concentration was 650 pM. Sequencing was performed on an Illumina NextSeq2000 for Paired End 50bp reads.

Computational methods

All pipelines were implemented in Snakemake version 6.11.0 [10] and used the same reference genome, a concatenated fasta file of GRCh38 [11] and GRCm38 [12] included in the cellranger software [13]. The cellranger human/mouse transcript annotation was used across all pipelines. The general workflow for the pipelines consists first of adapter trimming with cutadapt [14], and afterwards mapping and read counting with STARsolo [15]. Only when required by the different sequencing technologies parameters vary. This includes e.g. the treatment of UMIs, which were only present in Smart-seq3, or the parameters for the different locations of the barcodes (if present). It was ensured that all pipelines run the same version of all included programs.

PlexWell/FLASH-seq

All read files were trimmed with cutadapt version 3.6 [14] for 3' adapters with the –a option and a minimum length of 25 bp. In case of paired-end data, both reads were trimmed together, with the additional option –A (3' adapters for the second read). If samples were sequenced during more than a single sequencing run, then samples were concatenated after trimming for further processing. Before the processing with star, all reads were further concatenated into a single file. Reads were mapped with star version 2.7.9a [15], with the parameters --soloType SmartSeq --soloStrand Unstranded --soloUMIdedup Exact --outSAMtype BAM SortedByCoordinate --outSAMattributes NH HI AS nM. The parameter "Exact" for –soloUMIdedup was chosen, as this recapitulates the filtering for optical mapping, and is recommend for these technologies [16]. Reads per cell were counted to obtain statistics for downstream processing, and this was performed after mapping to account for losses during trimming. A custom script was used to parse the SAM file and count the mapped and unmapped reads. The conversion of SAM/BAM files and obtaining of related statistics was performed with Samtools version 1.11 [17]. Reads with the flags 0x4, 0x100 and 0x800 were ignored. Secondary alignments were excluded for the coverage graphs with the flag -F 256.

Smart-seq3

Cutadapt was used to separate pairs containing umi reads or not containing umi reads (body reads). Reads had to overlap with at least 8 bases of the 11 bp tag ATTGCGCAATG before the umi to be considered a umi read. Non-trimmed reads (body reads) were retained in a separate file.

Body reads were processed in the same way as in the PlexWell pipeline (including further trimming).

The following section only applies to the reads with UMIs. The umis were trimmed with cutadapt and a minimum overlap of 8 (option –O) to the full umis (11 bp tag, 8 bp degenerate

bases represented as N, 3 x G). Reads which did not satisfy this requirement, as e.g. the tag was detected at the end of a read and no 8 random bases with an addition of 3 x G could be matched, were discarded. . UMIs were retained via the --wildcard-file option. These UMIs were converted into a fastq file using a custom script. The umi reads were trimmed together with the umis with cutadapt for 3' adapters with the –a option, the "–pair-filter first" option and a minimum length of 25 bp. All reads and umis were concatenated into separate files. Afterwards the reads were mapped with star with the following options: --soloType CB_UMI_Simple –soloCBwhitelist None –soloCBstart 1 –soloCBlen 16 –soloUMIstart 20 –soloUMIlen 8 –soloBarcodeReadLength 0 –outSAMtype BAM SortedByCoordinate –outSAMattributes NH HI nM AS CR UR CB UB GX GN sS sQ sM. Read counts per cell were obtained in the same way as in the PlexWell workflow.

10x

The adapters were trimmed with cutadapt with the paired-end setting from the R2 read from the 3' end. All reads were concatenated into a single file. Star was run with the same parameters as for Smart-seq3, besides the options –soloUMIstart 17 –soloUMIlen 10. Read counts per cell were obtained in the same was as for the other workflows.

SORT-seq/VASA-seq

The pipeline for the SORT- and VASA-seq data was adapted from the 10X pipeline. The adapters were trimmed with cutadapt with the paired-end setting from the R2 read from the 3' end. Reads had to have a minimum length of 16 bp. All reads were concatenated into a single file. Star was run with the same parameters as for Smart-seq3, besides the options –soloType CB_UMI_Simple –soloCBstart 7 –soloCBlen 8 –soloUMIstart 1 –soloUMIlen 6 –soloBarcodeReadLength 26. Read counts per cell were obtained in the same way as for the other workflows.

HIVE

The pipeline for the HIVE and HIVE CLX data was adapted from the 10X pipeline. The adapters were trimmed with cutadapt with the paired-end setting from the R2 read from the 3' end, and reads had to have a minimum length of 26bp. All reads were concatenated into a single file. Star was run with the same parameters as for Smart-seq3, besides the options –soloCBstart 1, --soloCBlen 12, --soloUMIstart 13, --soloUMIlen 14 and –soloBarcodeReadLength 26. Read counts per cell were obtained in the same was as for the other workflows.

Evercode WT Mini

The pipeline for the Evercode WT Mini data was adapted from the 10X pipeline. The adapters were trimmed with cutadapt with the paired-end setting from the R2 read from the 3' end. Reads had to have a minimum length of 25 bp. All reads were concatenated into a single file. Star was run with the same parameters as for Smart-seq3, besides the options –soloType CB_UMI_Complex, --soloCBposition 0_10_0_17 0_48_0_55 0_78_0_85, --soloUMIposition 0_0_0_9 and –soloCBmatchWLtype 1MM. The barcodes were provided as whitelist. After the

processing the poly-A-barcodes and random hexamer barcodes belonging to a single well were identified, and profiles from both barcodes were merged into a combined profile for each single cell, based on the raw, unfiltered data. Afterwards a filtering as implemented in Star was performed on the combined data.

Bulk RNAseq

For experimental purposes, the bulk RNAseq data was processed with the same pipeline was PlexWell/FLASH-seq

Normalization

We choose to normalize the datasets per data-set and not per cell. In this way we retain as many characteristics as possible of the sequencing data sets to better represent what actual users would receive from their experiment. We normalized the data of each dataset to 20000 clusters per cell on average.

This amount was chosen based on the recommendation of 10X[18] and this has become a frequently used amount in the field. Previous studies have shown that 10000 is the lower limit to not lose any information [19].

Depending on the input file the normalization was implemented in two ways. If the input files were demultiplexed into different wells (Smart-seq3, Plexwell, FLASH-seq), the following algorithm was used. The total amount of cells before and after filtering were extracted, as well as the total amount of clusters (meaning in case paired-end reads one pair, in case of single-end reads one read) before any filtering steps. The amount of passing cells was then multiplied by 20.000 and divided by the total number of clusters, to get a scaling factor to adjust the input data amount. Next, this scaling factor was adjusted to account for data loss during the trimming step. The adjusted scaling factor was then applied to every input fastq file, to scale down the number of lines in the fastq file to a number divisible by four.

If the input files were not demultiplexed into different wells/barcodes (SORT-seq, VASA-seq, 10X, HIVE, HIVE CLX, Evercode WT Mini), the following implementation was used to calculate the scaling factor. The total amount of cells before and after filtering were extracted, as well as the total amount of clusters before any filtering steps. Next, the ratio of clusters assigned to cells passing the filtering and cells not passing the filtering was calculated. The amount of passing cells was then multiplied by 20.000 and divided by the total number of clusters, to get a scaling factor to adjust the input data amount. This factor was then first corrected for the ratio of reads within cells, and was furthermore adjusted to account for data loss during the trimming step. This adjusted scaling factor was then applied to every input fastq file, to scale down the number of lines to a number divisible by four.

It was not possible to normalize the mESC data obtained from the Evercode WT Mini kit, as well as the UMI part of the Smart-seq3 SR dataset, as not enough data has been generated. The full dataset was in this case used for further processing.

Regression analysis

The calculation of the relationships reads to detected features was performed with the smoothers_lowess function from the python statsmodels package version 0.14 [20], with the parameter frac set to 0.2. The input was the amount of reads and features per cell, all datasets per method combined. The averages of 10 times random subsampling to 90% of the dataset was used.

Ranking of not detected genes

To quantify how many genes were detected in bulk, but not detected in single cell methods (and vice versa), the following procedure was applied. Gene expression was ranked per method with the rankdata method from scipy version 1.5.3, with the method set to “ordinal”, and then sorted. Afterwards, 100 bins were created (ranked lowest to highest), and it was counted how many genes, which were not detected by the other method, were cumulatively present in these bins. The AUC was calculated with the “auc” method from sklearn. To detect a relationship between the amount of overlapping genes and non-detected genes, the pearson correlation between the AUC and the amount of non-detected genes was calculated.

Correlation analysis

To calculate the correlations for figure 10, all cells in a dataset were separated based on organism and then combined and treated as a single cell. Pearson correlation was calculated with scipy version 1.5.3.

Single cell analysis

The data was analyzed in R 4.2.1 [21] with Seurat 4.3.0 [22]. Data was log-normalized with a scaling factor of 10000. Before clustering, the data was filtered to retain only cells with more than 200 features, and with less than 20% human and mitochondrial mtRNA reads. The FindVariableFeatures function was executed with 2000 features. The value n.neighbors in the UMAP function was set to 11, to account for the smallest expected cluster (23 cells). All other values were set to default, and the workflow of the “guided tutorial” was followed [23].

Supplementary results and discussion

Return of cells

The amount of successfully sequenced cells on a plate or in a batch is a critical factor, but this investigation was not set up to systematically investigate this detail. E.g. the three plates for SORT- and VASA-seq were prepared, shipped and processed at the same time, which leads to potentially a bias in the outcomes for these methods. The HIVE method was tested the first time for this investigation, and its outcomes are probably suboptimal. In contrast to these points, the FLASH-seq method has been performed multiple times in our laboratory, and the success rate for this method is most likely higher. Despite these shortcomings, we still decided

to report some of these numbers. The datasets of the established methods (4 methods, 10 datasets), which were performed in-house, had a return of at least 83% of the cells, with one FLASH-seq dataset being an exception (48%). The SORT- and VASA-seq plates, which had to be shipped and were performed only once, had a return of 53-60% of the cells. Due to this setup it was not possible to evalute what the cause of this diminished return is. The three processed HIVEs returned only 8-11% of the expected cells, although the amount of duplicates indicated an appropriate loading of the HIVEs. This method was performed the first time, and according to the manufacturer a lower return can be expected in this situation.

Overall, the amount of returned cells cannot be properly evaluated in this setup, but there we can conclude that well-established methods should yield at least 80% of the expected cells.

Supplementary Table S1: Datasets in this study. In total 21 datasets were used in this study (1 x 10X, 3 x Smart-seq3, 4 x FLASH-seq, 2 x Plexwell, 3 x SORT-seq, 3 x VASAseq, 3 x HIVE, and 2 different bulk RNAseq sets with 3 replicates). Further columns include if the dataset contained human, mouse, or both cells, if the dataset was sequenced in paired-end or single-end mode, the respective read length and used sequencing machine, as well as further statistics.

Supplementary Table S2: Metrics per method**.** Minima, maxima and averages are provided separated for mESC and K562 cells, for features, diversity and mtRNA content.

Supplementary Table S3: Standard deviation in exon coverage per dataset, including the number of exons which had read coverage and were used for the calculations.

Supplementary Figure S1: FLASH-seq, comparison normalized versus non-normalized reads. The clustering shows one human cluster on the left, and three mouse clusters, separated by batch, on the right. Dataset number two did not contain any mouse cells. In all three mouse clusters the normalized and non-normalized reads group together. In the human cluster, cells are intermixed, with a slight gradient. Normalized and non-normalized datasets group approximately together.

Supplementary Figure S2: Smart-seq3, comparison normalized versus non-normalized reads. To compare the effect of the normalization, the normalized and non-normalized results of the Smart-seq3 umi datasets were compared. While the general clustering into species is still visibile (A), there is a separation into normalized and non-normalized samples (visible). Marked in orange are two sections were two normalized datasets group together, where their non-normalized counterparts cluster directly above.

1. Neagu, A., et al., *In vitro capture and characterization of embryonic rosette-stage pluripotency between naive and primed states.* Nat Cell Biol, 2020. **22**(5): p. 534-545.
2. Hagemann-Jensen, M., et al., *Single-cell RNA counting at allele and isoform resolution using Smart-seq3.* Nat Biotechnol, 2020. **38**(6): p. 708-714.

3. Hagemann-Jensen, M., et al., *Smart-seq3 Protocol V.3* Protocols.io, 2022.
4. Hahaut, V., et al., *Fast and highly sensitive full-length single-cell RNA sequencing using FLASH-seq.* Nat Biotechnol, 2022. **40**(10): p. 1447-1451.
5. Picelli, S. and V. Hahaut. *FLASH-seq protocol V.3* 2022 23.03.2022 [cited 2023 20.6]; Available from: https://www.protocols.io/view/flash-seq-protocol-b6myrc7w.html.
6. Muraro, M.J., et al., *A Single-Cell Transcriptome Atlas of the Human Pancreas.* Cell Syst, 2016. **3**(4): p. 385-394 e3.
7. van den Brink, S.C., et al., *Single-cell sequencing reveals dissociation-induced gene expression in tissue subpopulations.* Nature Methods, 2017. **14**(10): p. 935-936.
8. Hashimshony, T., et al., *CEL-Seq2: sensitive highly-multiplexed single-cell RNA-Seq.* Genome Biol, 2016. **17**: p. 77.
9. Salmen, F., et al., *High-throughput total RNA sequencing in single cells using VASA-seq.* Nat Biotechnol, 2022. **40**(12): p. 1780-1793.
10. Molder, F., et al., *Sustainable data analysis with Snakemake.* F1000Res, 2021. **10**: p. 33.
11. Schneider, V.A., et al., *Evaluation of GRCh38 and de novo haploid genome assemblies demonstrates the enduring quality of the reference assembly.* Genome Res, 2017. **27**(5): p. 849-864.
12. Church, D.M., et al., *Lineage-specific biology revealed by a finished genome assembly of the mouse.* PLoS Biol, 2009. **7**(5): p. e1000112.
13. Zheng, G.X., et al., *Massively parallel digital transcriptional profiling of single cells.* Nat Commun, 2017. **8**: p. 14049.
14. Martin, M., *Cutadapt removes adapter sequences from high-throughput sequencing reads.* EMBnet journal; Vol 17, No 1: Next Generation Sequencing Data AnalysisDO - 10 14806/ej 17 1 200, 2011.
15. Dobin, A., et al., *STAR: ultrafast universal RNA-seq aligner.* Bioinformatics, 2013. **29**(1): p. 15-21.
16. Dobin, A. *STARsolo: mapping, demultiplexing and quantification for single cell RNA-seq*. 2021 05.05.2021 [cited 2023 02.12.2023]; Available from: https://github.com/alexdobin/STAR/blob/master/docs/STARsolo.md#plate-based-smart-seq-scrna-seq.
17. Li, H., et al., *The Sequence Alignment/Map format and SAMtools.* Bioinformatics, 2009. **25**(16): p. 2078-9.
18. 10XGenomics. *What is the recommended sequencing depth for Single Cell 3' and 5' Gene Expression libraries?* 2018 [cited 2023 02.12.2023]; Available from: https://kb.10xgenomics.com/hc/en-us/articles/115002022743-What-is-the-recommended-sequencing-depth-for-Single-Cell-3-and-5-Gene-Expression-libraries-.
19. Pollen, A.A., et al., *Low-coverage single-cell mRNA sequencing reveals cellular heterogeneity and activated signaling pathways in developing cerebral cortex.* Nature Biotechnology, 2014. **32**(10): p. 1053-1058.
20. Seabold, S. and J. Perktold. *statsmodels: Econometric and statistical modeling with python*. in *9th Python in Science Conference*. 2010.
21. Team, R.C., *R: A Language and Environment for Statistical Computing*. 2022, Vienna, Austria: R Foundation for Statistical Computing.
22. Hao, Y., et al., *Integrated analysis of multimodal single-cell data.* Cell, 2021. **184**(13): p. 3573-3587 e29.
23. Laboratory, S. *Guided tutorial — 2,700 PBMCs*. 2023 27.03.2023 [cited 2023 21.07.2023]; Available from: https://satijalab.org/seurat/articles/pbmc3k_tutorial.html.
